# Annotations

**Published:** 1887-09-17

**Authors:** 


### ANNOTATIONS.

Medical Students.

THE Winter Session at the medical schools is fast approaching. The youth who enters upon the study of medicine launches his barque on an unknown sea. He may ultimately land on the shores of an honorary hospital physicianship, or on the strand of a fourth-rate parish appointment. It is possible that the voyage may come to a miserable end on the rocks of a chronic studentship or a permanent unqualified assistancy. We do not pity the sober and diligent youth who chooses medicine as a profession. He will in most cases find genuine pleasure in the new world of science to which his first two years of study will introduce him. Botany, zoology, chemistry, anatomy, physiology—these are studies which at the outset make many a weary brain; but as they are more deeply entered into and understood, not merely in their own spheres, but in the bearings they have upon all knowledge and all the problems which vex and yet ennoble the soul of man, they become fascinating and inspiring to an indescribable degree. The true student need never fear disappointment during the period of his scientific and professional studies. But very few men are students by nature; most require to be made so by circumstances. And it is the many bright and volatile youths who enter upon what to one who is not naturally studious is the most tedious of all labours for whom our pity and anxious sympathy are awakened. If they could but make up their minds to endure the drudgery of the first twelve months: to resist the temptation to seek change in questionable amusements and positive dissipations for that brief period, they would in most cases, by that time, have become so interested in their work that the keen edge of temptation would be blunted and almost powerless to harm. If any such read this little note, let them take a word of counsel from one who has travelled the way they are going, and by everything they hold most sacred and dear let them resolve that for one year at least they will spend all their time as they would if they were still under the eye of an anxious father and a loving mother at home. This one year may make them or ruin them for ever.

Medical Teachers.

Many a man has made shipwreck of a fair career who would have been an ornament to his calling if some brotherly and experienced counsellor had taken him by the hand in the days of his early manhood. The born teacher is rare, and many who are in the position of teachers have sometimes so little understanding of the magnitude of their responsibilities that they take no pains to supplement their own natural deficiencies. Too often, indeed, they are not conscious of any deficiencies at all. The work of a medical man is of a peculiarly delicate and almost sacred character; and those who do it should be trained in the rarest of schools. We are not going to say that they are not so trained. On the contrary, if our experience be a fair sample of that of ordinary medical men, there is no training better fitted to develop the highest qualities of mind and heart than that of the lecture-room and the hospital wards. But some teachers either do not care to remember their own student days, or are so naturally coarse in fibre that they not only acquire no influence for good over their pupils, but rather make both the science and practice of their art vulgar, commonplace, and altogether unlike the ideal work it should be. No man wishes to encourage prigs and pedants, but everyone who has spent years in the exercise of the delicate and difficult duties of a medical man longs to see those who are to undertake the same duties trained, taught, and inspired to aim at the very highest ideals both of manhood and professional duty. In view of the great responsibilities which are laid upon him no teacher can be offended if he be urged to try constantly to put himself in the place of his pupil, and also in the place of those to whom his pupil will ultimately be a trusted friend and helper, or a blind and worthless guide.

Medical Examiners.

Many medical students will hardly believe it, nevertheless it is quite true that examiners are men. What is more, they were once students who had to prepare for their "first professional" or their "first college." And some of them have even been plucked. What a consoling thought that must be to a young man who has just looked at the pass-list and failed to find his own name on it! If the invocations of deserving young men who have been shamefully treated by the examiners could have any influence on the spirits of the "vasty deep," the earthly pilgrimage of many respectable fathers of families would soon terminate in a catastrophe which it would require a Dante adequately to sing. The prison of examiners who should be tried by a "plucked" jury would have inscribed over its door in letters of fire, "All hope abandon ye who enter here." Nobody sympathises with the man who is "ploughed," or at least very few. Nevertheless, examinations are not always infallible tests of professional and scientific merit. Not seldom the reckless and impudent fellow, who has wasted three-fourths of the session in riotous idleness, pays a "coach," who "grinds him up" and "crams him" with "tips," with such sinister skill that he comes out with flying colours, whilst the conscientious but sometimes nervous man, who has worked hard early and late, may be too nervous to answer at all, so fearful is he of answering wrongly—he who may have scorned "tips" and "cramming," will sometimes come out a failure. Who is to blame for that? Too often the unskilful and even ignorant examiner. If examinations must be conducted as they are, those who appoint the examiners should feel it a serious duty to appoint only those who are intelligently fit for their work; and examiners should remember the golden rule, and examine others as they would wish to be examined themselves. The examiners examine the students; but who examines the examiners?

